# CRISPR/Cas9-Induced Mutagenesis of *TMS5* Confers Thermosensitive Genic Male Sterility by Influencing Protein Expression in Rice (*Oryza sativa* L.)

**DOI:** 10.3390/ijms23158354

**Published:** 2022-07-28

**Authors:** Yaoyu Fang, Jinlian Yang, Xinying Guo, Yufen Qin, Hai Zhou, Shanyue Liao, Fang Liu, Baoxiang Qin, Chuxiong Zhuang, Rongbai Li

**Affiliations:** 1 State Key Laboratory for Conservation and Utilization of Subtropical Agro-Bioresources, College of Agriculture, Guangxi University, Nanning 530004, China; flyfyyfyy@126.com (Y.F.); yangjinlian05@163.com (J.Y.); guoxinying24@126.com (X.G.); qyf15240680489@163.com (Y.Q.); liaosy26@163.com (S.L.); liufang1975@163.com (F.L.); bxqin@gxu.edu.cn (B.Q.); 2 State Key Laboratory for Conservation and Utilization of Subtropical Agricultural Bioresources, South China Agricultural University, Guangzhou 510642, China; haizhou@scau.edu.cn

**Keywords:** rice, CRISPR/Cas9, TGMS, *tms5*, proteome, two-line hybrid, grain quality

## Abstract

The development of thermosensitive genic male sterile (TGMS) lines is the key to breeding two-line hybrid rice, which has been widely applied in China to increase grain yield. CRISPR/Cas9 has been widely used in genome editing to create novel mutants in rice. In the present study, a super grain quality line, GXU 47, was used to generate a new TGMS line with specific mutations in a major TGMS gene *tms5* generated with CRISPR/Cas9-mediated genome editing in order to improve the rice quality of two-line hybrids. A mutagenesis efficiency level of 75% was achieved, and three homozygous T-DNA-free mutant lines were screened out. The mutants exhibited excellent thermosensitive male fertility transformation characteristics with complete male sterility at ≥24 °C and desirable male fertility at around 21 °C. Proteomic analysis based on isobaric tags for relative and absolute quantification (iTRAQ) was performed to unveil the subsequent proteomic changes. A total of 192 differentially expressed proteins (DEPs), including 35 upregulated and 157 downregulated, were found. Gene ontology (GO) analysis revealed that the DEPs were involved in a single-organism biosynthetic process, a single-organism metabolic process, oxidoreductase activity, and catalytic activity. Kyoto Encyclopedia of Genes and Genomes (KEGG) analysis showed that the DEPs were involved in ubiquinone and other terpenoid quinone biosynthesis, the biosynthesis of secondary metabolites, metabolic pathways, and phenylpropanoid biosynthesis. Our study shows that high mutation efficiency was achieved in both target sites, and T-DNA-free mutant lines were obtained in the T_1_ generation. The present study results prove that it is feasible and efficient to generate an excellent mutant line with CRISPR/Cas9, which provides a novel molecular mechanism of male sterility caused by the mutation of *tms5*.

## 1. Introduction

Rice (*Oryza sativa* L.) is one of the most important food crops in the world and a key component of the global food supply. With the persistent growth in the world’s population, the demand for food is increasing. Due to its higher yield than conventional rice, hybrid rice has been widely used and plays a vital role in increasing crop yield. Meanwhile, it has been cultivated in more than 40 countries and planted in 57% of the rice paddy fields in China [1,2,3]. Hybrid rice breeding includes a three-line and a two-line system [4]. The three-line system, including cytoplasmic male sterile (CMS) lines, restorer lines, and maintainer lines, is utilized to produce high-quality hybrid seeds and maintain the CMS line for sustainable production [5]. The restorer lines, with specific CMS-restorer genes, need to restore the fertility of the CMS lines. The three-line system has been the main type of hybrid rice, but its development is still limited because only a few germplasm resources of restorer lines can be used due to the genetic diversity between restorer lines and CMS lines [6]. Compared to the three-line system, the two-line system replaces CMS lines with photoperiod-sensitive genic male sterile (PGMS) or thermosensitive genic male sterile (TGMS) lines as the effective solution. TGMS lines or PGMS lines serve as sterility lines under restrictive conditions (high temperatures for TGMS and long days for PGMS) or maintainer lines under permissive conditions (low temperatures for TGMS and short days for PGMS) [7,8]. Genes controlling TGMS and PGMS are nuclear and recessive so that most of the normal rice varieties can restore male fertility in the PGMS and TGMS lines, providing a wider range of genetic resources for rice breeding [7,9,10,11]. Compared to the three-line system, the two-line system, due to its broad use of restorers, has an essential advantage in terms of labor- and time-saving, grain quality, crop yields, breeding efficiency, and economic benefits. Therefore, despite its relatively late development, it has occupied about one-third of the total area planted with hybrid rice in China [12].

In the last few years, many genes controlling the PGMS or TGMS trait have been identified and cloned. *tms5* is a nuclear recessive gene that controls the TGMS trait in AnnongS-1, which was the first spontaneously mutated indica rice (*Oryza sativa* ssp. *indica*) to be discovered in 1987. The *tms5*-determined TGMS lines have been utilized extensively in two-line hybrid rice breeding. *tms5* encodes the endonuclease RNase Z^S1^, which downgrades the temperature-sensitive *ubiquitin fusion ribosomal protein L40 (Ub_L40_)* mRNA to control the TGMS trait [11].

The clustered regularly interspaced short palindromic repeat (CRISPR)-associated protein 9 (Cas9) gene-editing technology is growing rapidly due to its simplicity and ease of use. There are a growing number of examples of its use in crop trait improvement. Meanwhile, with the ability to precisely edit plant gene sequences, the application of CRISPR/Cas9 technology in agriculture will be of great value [13,14]. It has successfully improved crop quality, abiotic stress tolerance, and yield [15,16,17,18,19,20,21]. In addition, CRISPR/Cas9 technology has made it possible to edit multiple genes at the same time [22].

Proteomic analysis has proven to be a powerful tool for detecting protein changes caused by mutations. With the dramatic advancement in bioassay tools, iTRAQ (isobaric tags for relative and absolute quantification)-based proteomic analysis provides a novel way to quantitatively analyze protein abundance expression patterns and underlying molecular mechanisms for mutagenesis [23,24,25].

Despite the presence of many effective experimental methods and well-proven prediction tools, the vastly complex mechanisms leading to genetic mutations remain to be revealed. A few studies have been carried out related to thermosensitive genic male sterility, but there have been no iTRAQ-based proteomics studies to understand the thermosensitive genic male sterility induced by CRISPR/Cas9. In this research, we generated TGMS mutants with *tms5* and applied proteomics to analyze the results of CRISPR-induced TGMS mutant events. Our study provides some new evidence supporting the TGMS regulatory module in rice.

## 2. Results

### 2.1. Construction of CRISPR/Cas9 Knockout Vector

**Figure 1:**
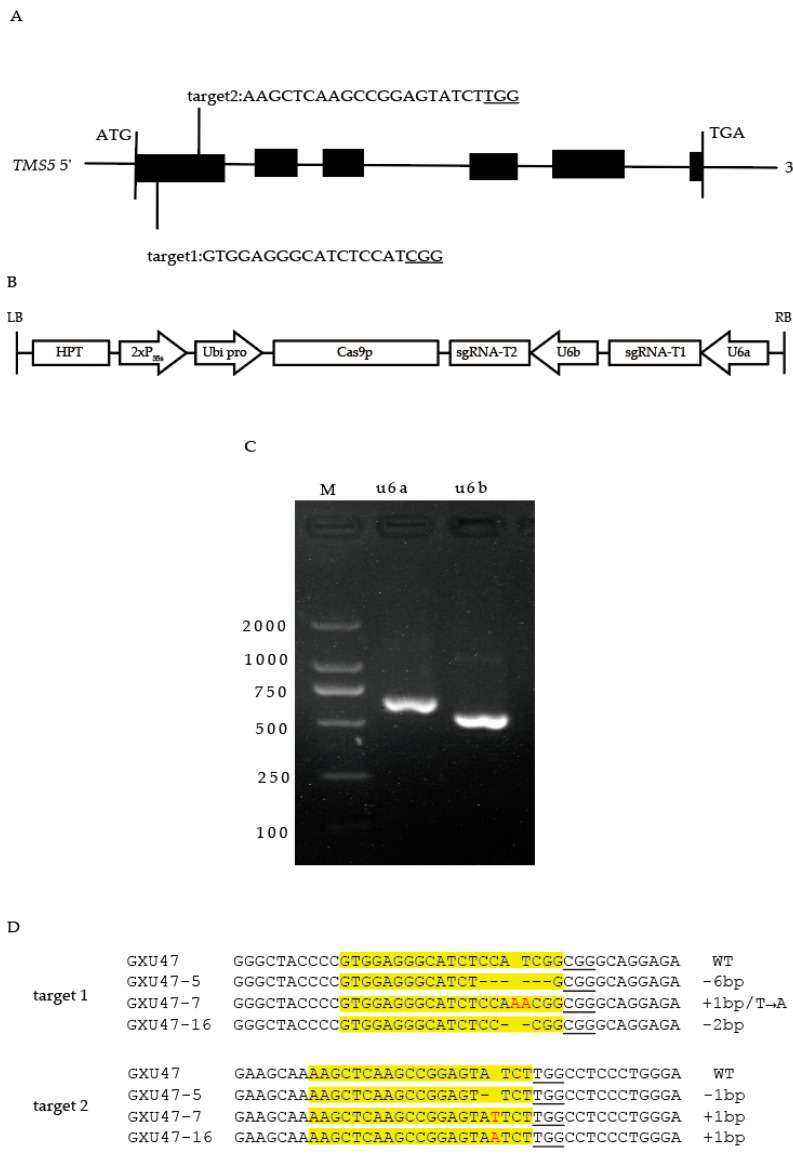
(**A**) Schematic diagram of the location of targeted sites in *tms5*. (**B**) Schematic diagram of the CRISPR/Cas9 knockout vector. (**C**) sgRNA expression cassettes for two targets of u6a and u6b. (**D**) Sequencing results of targeted regions of *tms5* in three T_1_ transgenic plants. Target sequences are shown in yellow. PAMs are underlined. Insertions and inserted nucleotides are marked in red. Deleted nucleotides are shown as dashed lines.

Based on the *tms5* sequence of GXU47 obtained by sequencing, two targets were determined on CRISPR-GE (http://skl.scau.edu.cn/ (accessed on 9 July 2018)), both of which were on the first exon (Figure 1A). The sgRNA expression cassettes for two targets were amplified via overlapping PCR (Figure 1C). The purified amplification products and pYLCRISPR/Cas9Pubi-H vector cleaved by Bsa I restriction endonuclease were ligated by T_4_ DNA ligase. The CRISPR/Cas9 knockout vector was successfully constructed (Figure 1B), and the sequences of the sgRNA expression cassettes were confirmed to be correct using the specific primers (Table S1).

### 2.2. Identification of Transgenic Positive Plants and Sequencing of Target Sites

Specific detection primers (Table S1) were utilized to identify the positive mutant lines after the CRISPR/Cas9 vector was transformed into GXU47 embryogenic calli. A total of 16 plants were obtained in the T_0_ generation, including 12 positive plants. There were six homozygous, five heterozygous, and one WT plant at the first target site, and five homozygous and seven heterozygous plants at the second target site. Homozygous mutations were detected in both of the two targets of the three mutant lines (GXU47-5, 7, and 16). Specific primers were designed for the four most probable off-target sites for each target and then used for PCR amplification and sequencing (Table S1). The results show that no off-target mutations were detected.

### 2.3. Identification of T-DNA-Free Plants

Tracing the segregation of T-DNA in the T_1_ generation derived from T_0_ plants, 16 T-DNA-free plants were obtained. Among them, three homozygous mutant plants, GXU47-5-1, GXU47-7-5, and GXU47-16-2, were selected for further investigations. GXU47-5-1 showed homozygous mutations with 6 and 1 bp deletions on the first and second target sites, respectively. Homozygous mutant GXU47-7-5 showed a 1 bp substitution and a 1 bp insertion on the first target site and a 1 bp insertion on the second target site. The mutant line GXU47-16-2 was a homozygous mutant with a 2 bp deletion on the first target site and a 3 bp insertion on the second target site (Figure 1D).

### 2.4. Transformation of Fertility in the Mutants

**Figure 2:**
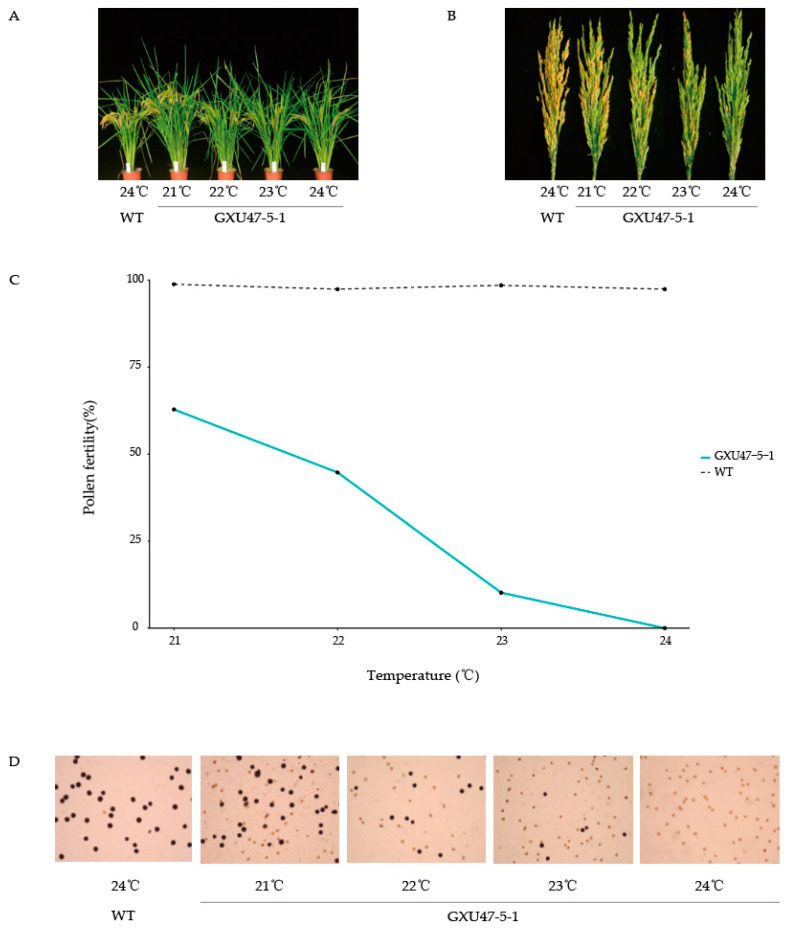
Male fertility transformation of line GXU47-5-1. (**A**) Plant morphologies of WT and GXU47-5-1 at different temperatures. (**B**) Change in spikelet fertilities in panicles of WT and GXU47-5-1 along with changes in temperature. (**C**) Trend of pollen fertility rates with changes in temperatures. (**D**) Observation of pollen fertility rates of WT and GXU47-5-1 at different temperatures under a microscope.

The pollen fertility rates of the mutant line GXU47-5-1 were 62.8%, 44.7%, 10.2%, and 0% at a constant light intensity of 4000 Lx and temperatures of 21, 22, 23, and 24 °C, respectively, showing a clear downward trend with the decreasing temperature between 21 and 24 °C, while the pollen fertility rates of the wild-type GXU47 were 98.8%, 97.4%, 98.5%, and 97.4%, at the respective temperatures and were not affected by temperature (Figure 2D).

The spikelet fertility rates of the mutant line GXU47-5-1 were 42.7%, 22.5%, 7.5%, and 0%, showing a significant downward trend with the decreasing temperature between 21 and 24 °C, and reaching complete sterility at 24 °C, while the wild-type rates were 80.6%, 81.8%, 77.5%, and 81.6%, showing no significant difference with the changes in temperature (Figure 2B). The results indicate that the GXU47-5-1 line presents a TGMS characteristic, and the male fertility transformation of the line occurred at 24 °C.

### 2.5. Main Agronomic Traits of Wild Type and Mutant Lines

The main agronomic traits of mutant line GXU47-5-1 (T_1_) and its WT type were measured. The results show non-significant differences in the panicle length (PL), 1000-grain weight (GWT), flag leaf length (FLL), and flag leaf width (FLW) between GXU47-5-1 (T_1_) and WT (Table 1). The GXU47-5-1 line presents a significant increase in the effective panicle number (EPN) and a significant decrease in the plant height (PH). The EPN increased from 8.52 to 12.44 cm, and the PH decreased from 91.21 to 82.50 cm. The main agronomic data of GXU47-5 in the T_2_ and T_3_ generations were also recorded. As expected, the mutant lines show significantly increased EPN and decreased PH, but there were no significant differences in the GWT, FLL, FLW, or PL. The results are consistent with the T_1_ generation, showing that the mutated characters were stably inherited.

### 2.6. Protein Identification and Quantitative Analysis of Male Sterile Mutants and Wild Type

Three replicate iTRAQ experiments were performed in 574,193 WT- and GXU47-5-1 generated spectra. In order to reduce the false positive rate, only peptide spectrum matches (PSMs) with a confidence level of more than 99% were trusted, and the proteins containing at least one unique peptide were treated as reliable proteins. Peptides and proteins with an FDR >1% were screened out. Finally, we identified 26,986 peptides corresponding to 4752 proteins. After screening with a minimum fold change of 1.5, a total of 192 DEPs were obtained, among which 35 were upregulated and 157 were downregulated. We manually searched among all the DEPs that are associated with thermosensitive genic male sterility and flower development (Table 2) and found that Q84UR8 (cold shock domain protein 2), Q6EQG6 (small heat shock protein), A2Y653 (ICE-like protease p20 domain-containing protein, putative), A0A0E0H559 (uncharacterized protein), and A2X9T6 (uncharacterized protein) were related to the response to temperature. Q84UR8 was upregulated, while Q6EQG6, A2Y653, A0A0E0H559, and A2X9T6 were downregulated in the mutant line. A0A0E0FJ22, A0A0E0G9Q7, A0A0E0FK01, and A0A0E0ITF3 were found to be related to RNA binding and were all downregulated in the mutant line. A0A0E0IIY5 (sugar transporter), A0A0E0GNW4 (sucrose synthase), A0A0E0GAC4 (sugar transporter), B8AIG0 (starch synthase), and A2YQL4 (fructokinase-2) were related to the synthesis and transport of starchy carbohydrates. All of them were downregulated in the mutant line.

### 2.7. GO and KEGG Pathway Enrichment Analysis Results of DEPs

**Figure 3:**
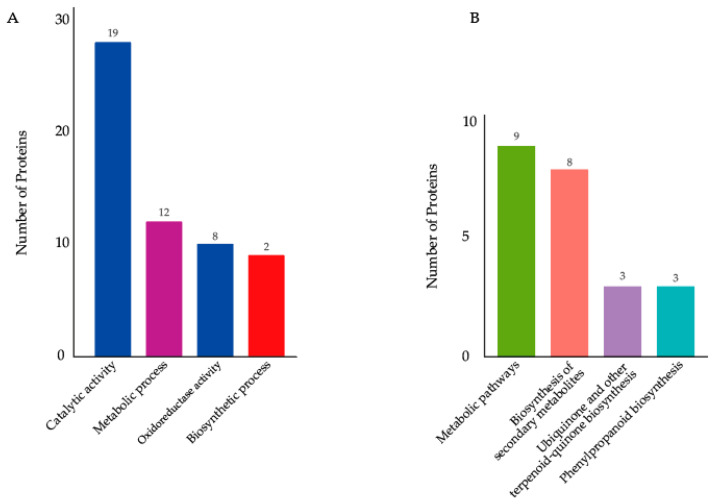
(**A**) Significantly enriched GO terms of differentially expressed proteins DEPs. (**B**) Significantly enriched KEGG pathways of DEPs.

Gene ontology (GO) and Kyoto Encyclopedia of Genes and Genomes (KEGG) enrichment analyses were performed on a set of DEPs, and their different functions were identified. The *p*-value was adjusted using multiple hypothesis tests, and the enriched terms were selected using the false discovery rate (FDR). The results of the GO enrichment analysis show that DEPs associated with the “biological process (BP)” were mainly related to the single-organism biosynthetic process and single-organism metabolic process. Regarding “molecular function (MF)”, oxidoreductase activity and catalytic activity were enriched (Figure 3A). The KEGG analysis revealed that the DEPs were enriched in ubiquinone and other terpenoid quinone biosynthesis, the biosynthesis of secondary metabolites, metabolic pathways, phenylpropanoid biosynthesis, phenylalanine metabolism, and phenylalanine, tyrosine, and tryptophan biosynthesis (Figure 3B).

### 2.8. RT-qPCR-Based Gene Expression Analysis and Validation of Proteomic Data

**Figure 4:**
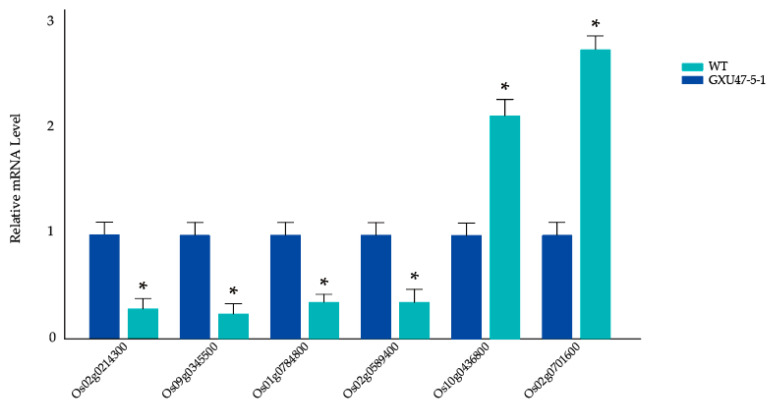
Relative expression levels of *TMS5 (Os02g0214300)* and responsive genes of five selected DEPs. WT is indicated in blue and GXU47-5-1 is indicated in cyan. Significant differences in the expression levels are indicated by “*”; Student’s *t*-test, *p* ≤ 0.05.

RT-qPCR was used to detect the different expression levels of the target gene *TMS5* (*Os02g0214300*) in mutant plants and WT. Meanwhile, to validate the proteomic data, five genes associated with DEPs were selected, two of which encode upregulated proteins, including A0A0P0XV01 (*Os10g0436800*) and Q6ZIK0 (*Os02g0701600*), and three of which encode downregulated proteins, including Q6EQG6 (*Os09g0345500*), Q8LQN2 (*Os01g0784800*), and Q6YY41 (*Os02g0589400*). The RT-qPCR results show that *TMS5* expression was significantly decreased in the mutant compared to in the WT, and the expression levels of the selected genes were consistent with the proteomics data (Figure 4).

### 2.9. Application of GXU47S in Hybrid Rice Breeding

**Figure 5:**
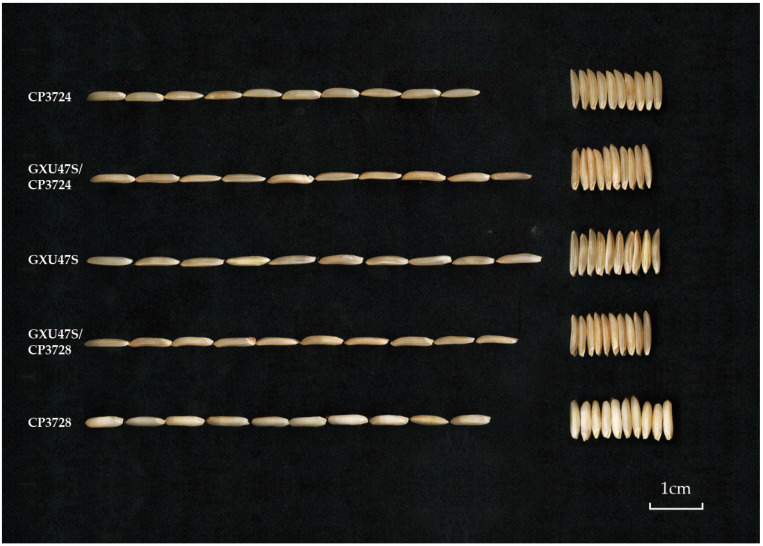
Grain phenotype of GXU47S/CP3724, GXU47S/CP3728, and their parents.

The obtained T-DNA-free TGMS line GXU47S was crossed with restorers, CP3724 and CP3728, respectively. The two-line hybrids are much slender with significantly longer grain length than the male parents and narrower grain width than both parents and most two-line hybrids. The grain length–width ratio of hybrids GXU47S/CP3724 and GXU47S/CP3728 reached 5.5 as compared 4.0 in both restorers and less than 3.6 in most two-line hybrids (Figure 5, Table 3). Three major chemical and cooking parameters which were strongly relative eating quality, gel consistency (GC), amylose content (AC) and 2-acetyl-1-1pyrroline (2-AP) content of the two hybrids were compared with their parents. Results showed excellent soft-rice quality parameters in both hybrids, GXU47S/CP3724 and GXU47S/CP3728, as with high GC, relative lower AC as well as high 2-AP content, which were similar as those of the female parent, GXU47S (Table 3).

## 3. Discussion

Thermosensitive genic male sterile (TGMS) lines have been widely applied in two-line hybrid rice breeding for more than 20 years. *tms5* is the main TGMS source and was found in more than 70% of the commercial TGMS lines developed in China. Compared to traditional breeding, genetic engineering in breeding has advantages, including time- and labor-saving and greater effectiveness. As a well-developed genetic engineering tool, the CRISPR/Cas9 system has been widely utilized to improve crop quality and/or increase yield in many plants [15,16,17,18,19,20,21]. It provides promising prospects to obtain mutants with desired traits. In the present work, TGMS mutant lines with *tms5* via CRISPR/Cas9 were generated successfully, and proteomic analysis was performed to elucidate the impacts of mutations on a whole-genome level. This work further enriches the new types of TGMS lines for the development of new two-line hybrid varieties in rice.

In the present study, TGMS mutant lines were successfully generated using the CRISPR/Cas9 gene-editing system, and homozygous and heterozygous plants were simultaneously obtained in the T_0_ generation. A total of 16 T_0_ plants were identified using Sanger sequencing, with 18.75% homozygous, 56.25% heterozygous, and 25% WT. The results show high genome editing efficiency with CRISPR/Cas9 in rice and a high probability of obtaining homozygous mutants in the T_0_ generation, which is consistent with the results of previous studies [20,26]. There was no off-target observed in the detection of the eight most likely sites, indicating that CRISPR/Cas9 has a very low off-target rate in rice. A previous study also showed that inherited Cas9 rarely induces off-target mutations [21]. Heritable homozygous TGMS plants are crucial to two-line hybrid rice breeding. Plants without T-DNA were screened out at a frequency of 35.6%. The same mutations of *TMS5* in the T_1_ and T_2_ generations indicate that homozygous mutants could be stably inherited in subsequent generations. Moreover, in our study, mutant lines showed a relatively low critical sterility-inducing temperature (CSIT) of 24 °C, indicating a lower and more stable CSIT in CRISPR/Cas9-mediated TGMS plants as compared to that in RNAi-mediated TGMS plants [27]. 

iTRAQ-based proteomic analysis was performed to identify the different expressions in the WT and mutant plants. A total of 4752 proteins were identified, including 192 DEPs, with 35 upregulated and 157 downregulated. Functional and pathway analyses were performed to reveal the potential molecular interactions among the different proteins.

The GO analysis showed that the DEPs were mainly enriched in the single-organism biosynthetic process, single-organism metabolic process, oxidoreductase activity, and catalytic activity. These four biological processes are the basis of plant growth and development, which can affect anther development and male fertility. In previous studies, *tms5* encoded the endonuclease RNase Z^S1^ which degraded the temperature-sensitive *ubiquitin fusion ribosomal protein L40 (Ub_L40_)* mRNA to control the TGMS trait [11]. *tms5* is related to 3′-tRNA processing endoribonuclease activity (which is involved in the biosynthetic process, metabolic process, and catalytic activity), tRNA 3′-end processing (which is involved in the biosynthetic process and metabolic process), and tRNA 3′-trailer endonucleolytic cleavage (which is involved in the biosynthetic process and metabolic process).

The KEGG analysis showed that DEPs were mostly enriched in ubiquinone and other terpenoid quinone biosynthesis, the biosynthesis of secondary metabolites, metabolic pathways, and phenylpropanoid biosynthesis. The biosynthesis of secondary metabolites and metabolic pathways, such as ubiquinone and other terpenoid quinone biosyntheses, have a general impact on plant fertility [28]. Phenylpropanoid biosynthesis also plays an important role in male sterility in a series of plant species [25,29,30,31]. Phenylpropanoid is the chemical composition of sporopollenin, which is a key component of the microspore and outer cell wall of pollen [31]. In addition, a number of genes in the biosynthesis of phenylpropanoid interact with MYB transcription factors involved in fertility conversion [30]. Therefore, phenylpropanoid biosynthesis plays an important role in the sterility caused by *tms5*.

The proteins Q84UR8 (cold shock domain protein 2), Q6EQG6 (small heat shock protein), A2Y653 (ICE-like protease p20 domain-containing protein, putative), A0A0E0H559 (uncharacterized protein), and A2X9T6 (uncharacterized protein) were found to be related to the response to temperature in this study. CSP2 is the negative regulator of cold acclimation, and upregulated CSP2 resulted in a decreased freezing tolerance and late flowering [32]. sHSPs play an important role in the response to heat stress and plant growth and development [33]. ICE was identified as an important factor in the low-temperature stress response [34]. Q84UR8 was upregulated, while Q6EQG6, A2Y653, A0A0E0H559, and A2X9T6 were downregulated in the mutant line. These results suggest that TGMS mutation leads to decreased heat tolerance. Male sterility under relatively higher temperatures may be related to these factors.

There were non-significant differences in the 1000-grain weight, flag leaf length, flag leaf width, and panicle length between the mutant lines and WT. The GXU47-5 line showed a significant increase in the effective panicle number and a decrease in plant height. The mutant lines can serve as an excellent male sterile line in rice breeding to reduce plant height and increase tillering. This study proves that it is feasible and efficient to generate an excellent mutant line using CRISPR/cas9.

In conclusion, the present study created new excellent T-DNA-free TGMS lines with Basmati background using the CRISPR/Cas9 system without the introduction of deleterious mutations, which could improve the current inferior quality of two-line hybrid rice. The results provide a novel molecular mechanism of male sterility caused by the *TMS5* mutation. In recent years, several studies have been reported on *TMS5*, but few iTRAQ-based proteomics analyses of CRISPR/Cas9-mediated *TMS5* mutants have been performed. The combination of CRISPR/Cas9 and proteomics can help explain the effect of gene mutation on the whole plant proteome, which can be widely applied in the future. Further studies could reveal the molecular mechanism of *tms5* leading to male sterility in rice to easily obtain excellent TGMS lines.

## 4. Materials and Methods

### 4.1. Plant Materials and Growing Environment

GXU47, an indica rice line, was used for genetic transformations. The line was developed at the Rice Research Institute of Guangxi University. It has a Basmati background with excellent agronomic traits and fragrance and has good potential to improve the inferior quality of the two-line hybrid rice. 

### 4.2. Construction of CRISPR/Cas9 Knockout Vectors

The sequence of *TMS5* of wild-type GXU47 was amplified by specific primers and then imported into the CRISPR-GE (http://skl.scau.edu.cn/ (accessed on 9 July 2018)) website to design targets. The targets generally require an off-target rate of less than 0.6 and a GC content between 45% and 70%. Two targets were selected, the first in exon 1 at 116–131 bp and the second in exon 2 at 623–642 bp. Overlapping PCR (method 2) was performed with specific primers to introduce two targets into the two sgRNA expression cassettes, respectively. U6a and U6b promoters were used to drive the sgRNA expression cassettes. The purified expression cassettes were mixed with the pYLCRISPR/Cas9P_ubi_-H plasmid to construct the knockout vector. The mixture was simultaneously digested by *Bsa* I restriction endonuclease and ligated by T4 ligase [22]. The vector was later transformed into the calli of GXU47 by Agrobacterium EHA105 [35]. The sequences of all the primers are listed in Table S1.

### 4.3. Detection of T_0_ Generation Genotypes and Screening for T-DNA-Free Plants

Genomic DNA was extracted from the fresh leaves of all T_0_ generation lines. Then, the Cas9 gene-specific primers (Cas9-F/Cas9-R) were utilized to detect the positive plants. The segregation of the T-DNA in the T_1_ generation derived from T_0_-positive plants was traced by amplifying Cas9 sequences to screen for T-DNA-free plants. The target sequences were amplified by specific primers, and the mutations were identified by Sanger sequencing. Four potential off-target sites of each target site were predicted using the CRISPR-GE (http://skl.scau.edu.cn/ (accessed on 6 November 2018)) website, and the detection primers (Table S1) were designed. The off-target sites were amplified by the detection primers and sequenced for analysis.

### 4.4. Characterization of Phenotypes

Plants of different generations were grown in standard greenhouse conditions (16 h light at 30 °C/8 h dark at 22 °C) for male sterility plant evaluation, and the identified male sterile plants were ratooned and grown in a pool irrigated with cool water at a constant temperature of 20 °C for seed multiplication to generate the next generation. Seedlings derived from T_1_ male sterile plants were grown in the pots, and when the plants grew to the early panicle developmental stage, the pots were moved into phytotrons at temperatures of 21, 22, 23, and 24 °C to test for the male fertility transformation characteristic of the plants. Pollen fertility was identified by staining mature pollen grains with 1% I2–5% KI solution and photographed using a Zeiss Scope A1 microscope.

### 4.5. Protein Extraction, Digestion, and Labeling

The total protein was extracted from young panicles from stages of microspore mother cell (MMC) to meiosis in wild-type (GXU47) and its mutant line (GXU47-5-1) grown at restrictive temperatures of 24 °C, with three replicates. The samples were minced individually with liquid nitrogen, and the proteins were extracted according to the methods described previously [36]. The protein solution for each sample was adjusted to contain exactly 0.12 mg of protein and digested with Trypsin Gold (Promega, Madison, WI, USA) at an enzyme-to-substrate ratio of 1:50. After digestion at 37 °C for 16 h, the peptides were desalted using a C18 cartridge and dried by vacuum centrifugation. The desalted peptides were labeled with iTRAQ reagent (iTRAQ^®^ Reagent-8PLEX Multiplex Kit, Sigma, St. Louis, MO, USA) according to the manufacturer’s instructions. The samples were labeled as (GXU47)-114, -115, and -116 and (GXU47-5)-117, -119, and -121. Further experimental procedures were carried out according to the previously established methods [37].

### 4.6. GO and KEGG Analysis

The proteins were identified using Proteome Discoverer 2.2. The database used for this study was UniProt Oryza sativa (FASTA) (215,502 sequences). By analysis of the protein expression differences, the differentially expressed proteins (DEPs) with a fold change greater than 1.5 were obtained. The DEP list was then uploaded to KOBAS (http://kobas.cbi.pku.edu.cn/kobas3/genelist/ (accessed on 16 March 2021)) for KEGG pathway enrichment analysis. The species option was selected as “Oryza sativa japonica (Japanese rice) (RefSeq)”, and the other options were defaulted. In addition, the DEPs were analyzed on the agriGO website (http://bioinfo.cau.edu.cn/agriGO/ (accessed on 16 March 2021)), with all the options set to default.

### 4.7. RT-qPCR and Validation of Proteomic Data

The total RNA was isolated from fresh leaves using Vazyme’s FastPure Plant Total RNA Isolation Kit. A total of 5 proteins were selected to verify the proteomic data using RT-qPCR. The rice actin gene served as the reference gene. The relative expression levels were assessed using the 2^−∆∆Ct^ method.

### 4.8. Agronomic Trait Characterization

The identified male sterile plants of different generations were grown in a pool irrigated with cool water with a constant temperature of 20 °C. Five plants of each strain were randomly investigated. The agronomic traits were characterized by measuring the plant height, effective panicle number, panicle length, flag leaf width, grain length, grain width (mm), and 1000-grain weight after the rice had reached maturity. The measurement of gel consistency, amylose content, and 2-acetyl-1-1pyrroline (2-AP) content was entrusted to Guangxi University Testing Company. 

## Figures and Tables

**Table 1:** Main agronomic traits of wild-type and homozygous mutant lines in the T_1_, T_2_, and T_3_ generations.

Generation	Line	PH (cm)	EPN	PL (cm)	FLL (cm)	FLW (cm)	GWT (g)
T_1_	WT	91.21 *	8.52 *	21.85 ^ns^	45.33 ^ns^	1.54 ^ns^	24.38 ^ns^
	GXU47-5-3	82.50 *	12.44 *	22.55 ^ns^	46.76 ^ns^	1.53 ^ns^	23.85 ^ns^
T_2_	WT	91.45 *	8.72 *	21.77 ^ns^	46.85 ^ns^	1.55 ^ns^	24.32 ^ns^
	GXU47-5-3-1	82.03 *	12.52 *	21.56 ^ns^	46.23 ^ns^	1.53 ^ns^	24.35 ^ns^
T_3_	WT	91.99 *	8.63 *	21.95 ^ns^	45.82 ^ns^	1.54 ^ns^	24.38 ^ns^
	GXU47-5-3-1-1	82.33 *	12.74 *	21.55 ^ns^	46.02 ^ns^	1.57 ^ns^	23.88 ^ns^

WT: wild type; PH: plant height; EPN: effective panicle number; PL: plant length; FLL: flag leaf length; FLW: flag leaf width; GWT; 1000-grain weight; * and ns represent a significant and non-significant difference, respectively. Student’s *t*-test, *p* ≤ 0.05. These data are the means of five independent samples from T_1_, T_2_, and T_3_ generations of GXU47-5.

**Table 2:** DEPS related to thermosensitive genic male sterility and flower development.

Protein	Annotation	Regulation
Q84UR8	cold shock domain protein 2	Up
Q6EQG6	small heat shock protein	Down
A2Y653	ICE-like protease p20 domain containing protein, putative	Down
A0A0E0H559	uncharacterized protein	Down
A2X9T6	uncharacterized protein	Down
A0A0E0FJ22	uncharacterized protein	Down
A0A0E0G9Q7	uncharacterized protein	Down
A0A0E0FK01	uncharacterized protein	Down
A0A0E0ITF3	uncharacterized protein	Down
A0A0E0IIY5	sugar transporter	Down
A0A0E0GNW4	sucrose synthase	Down
A0A0E0GAC4	sugar transporter	Down
B8AIG0	starch synthase	Down
A2YQL4	fructokinase-2	Down

**Table 3:** Quality traits of hybrids and their parents.

Line	Grain Length (mm)	Grain Width (mm)	Grain Length–Width Ratio	Gel Consistency (mm)	Amylose Content (%)	Content of 2-AP (μg/kg)
GX47S/CP3724	8.23 ± 0.27	1.46 ± 0.07	5.6 ± 0.3	72.1 ± 2.2	17.0 ± 0.8	930 ± 15
GXU47S	8.46 ± 0.45 ^ns^	1.63 ± 0.03 *	5.2 ± 0.2 ^ns^	76.5 ± 2.4 *	14.5 ± 0.3 *	964 ± 8 *
CP3724	7.34 ± 0.09 *	1.68 ± 0.03 *	4.4 ± 0.1 *	66.3 ± 1.9 *	18.3 ± 0.2 *	865 ± 9 *
GXU47S/CP3728	8.13 ± 0.38	1.48 ± 0.02	5.5 ± 0.2	71.8 ± 3.2	17.4 ± 0.5	922 ± 18
GXU47S	8.46 ± 0.45 *	1.63 ± 0.03 *	5.2 ± 0.2 ^ns^	76.5 ± 2.4 *	14.5 ± 0.3 *	964 ± 8 *
CP3728	7.62 ± 0.31 *	1.92 ± 0.10 *	4.0 ± 0.2 *	64.2 ± 2.0 *	18.0 ± 0.2 *	873 ± 6 *

* and ns represent a significant and non-significant difference, respectively. Student’s *t*-test, *p* ≤ 0.05.

## Supplementary Materials

The supporting information can be downloaded at: https://www.mdpi.com/article/10.3390/ijms23158354/s1.
