# Supplementary material for: CRISPR/Cas9-Induced Mutagenesis of TMS5 Confers Thermosensitive Genic Male Sterility by Influencing Protein Expression in Rice (Oryza sativa L.)
Source: Int J Mol Sci. 2022 Jul 28;23(15):8354. doi: 10.3390/ijms23158354 (PMC9369173; doi:10.3390/ijms23158354)
Supplement: Supplementary file 1 [file ijms-23-08354-s001.zip › ijms-1822198-supplementary.pdf]

Table S1. Primers used in this study

Primer	Primer sequence (5'→3')	For experiment
U-F	CTCCGTTTTACCTGTGGAATCG	Vector construct
gR-R	CTCCGTTTTACCTGTGGAATCG	Vector construct
gR-T1	TGGAGGGCATCTCCATCGGGTTTTAGAGCT AGAAAT	Vector construct
U6a-T1	CCGATGGAGATGCCCTCCACGGCAGCCAA GCCAGCA	Vector construct
gRT2	AAGCTCAAGCCAGAGTATCTGTTTTAGAGC TAGAAAT	Vector construct
U6b-T2	AGATACTCTGGCTTGAGCTTCAACACAAGC GGCAGC	Vector construct
Pps-R	TTCAGAGGTCTCTACCGACTAGTCACGCGT ATGGAATCGGCAGCAAA	Vector construct
Pgs-2	AGCGTGGGTCTCGTCAGGGTCCATCCACTC CAAGCTC	Vector construct
Pps-2	TTCAGAGGTCTCTCTGACACTGGAATCGGC AGCAAAGG	Vector construct
Pgs-L	AGCGTGGGTCTCGCTCGACGCGTATCCATC CACTCCAAGC	Vector construct
SP-L1	GCGGTGTCATCTATGTTACTAG	Vector sequencing
SP-R	TGCAATAACTTCGTATAGGCT	Vector sequencing
Cas9-f	CTGACGCTAACCTCGACAAG	Cas9 detection
Cas9-r	CCGATCTAGTAACATAGATGACACC	Cas9 detection
off-site1F	GACTCCAGGTGGGTTGGTTG	Off-site detection
off-site1R	TTTTTCTTCAGGTTCACCGGC	Off-site detection
off-site2F	TCAGTTGCCCCTCAACCTTC	Off-site detection
off-site2R	ACCTTGGCTTTAGTTGCGGT	Off-site detection
off-site3F	AGGCTGAGGTGATTTGGACG	Off-site detection
off-site3R	TGATCTGCCATCTGCCCAAG	Off-site detection
off-site4F	GCAGTACAGCCTTCGACCAT	Off-site detection
off-site4R	GCTAGCGGTAGTTGCAGACA	Off-site detection
off-site5F	CTCAGAGTTGCATAGGCGGT	Off-site detection
off-site5R	TCAAAATGTTCATGTGTCCCAGC	Off-site detection
off-site6F	CGTGTCGAGTTCCCTCAAGT	Off-site detection
off-site6R	TGGCACTGTCCATTGAACCT	Off-site detection
off-site7F	TGGCCGAGGAATGTGTCAAA	Off-site detection
off-site7R	TCGCTACCAACCGATGTTGT	Off-site detection
off-site8F	CGCCAAGAGCTTTGGTGATG	Off-site detection
off-site8R	GATTGGCTGGCCTGTTCTCT	Off-site detection
tms5(1)-F	CGACCTCCACCACCGC	Target 1 sequencing
tms5(1)-R	TTCCTCTTCATCTCCCACGC	Target 1 sequencing
tms5(2)-F	GTCCCCCTCGAGATTGGTCA	Target 2 sequencing
tms5(2)-R	GACGGTGCCTGAGATTGCT	Target 2 sequencing
Os10g0436800-F	GGAGACCACCGCTTGGAC	RT-qPCR
Os10g0436800-R	TTGAACATGCCTGACCCGAA	RT-qPCR
Os02g0701600-F	TGATGCATTGGAGCAGCCTT	RT-qPCR
Os02g0701600-R	CCAGCTCGCTTACAAACTGC	RT-qPCR

Primer	Primer sequence (5'→3')	For experiment
Os09g0345500-F	AGGAAGTCTCCGCGTCTTTT	RT-qPCR
Os09g0345500-R	TGCACCAGGGCAAAGGGT	RT-qPCR
Os01g0784800-F	GCGGTGAAGGTCTATGTCGT	RT-qPCR
Os01g0784800-R	AGAGTTTCAGGAACCTGCCAT	RT-qPCR
Os02g0589400-F	GGAATGGCCGAACAGATAAGAC	RT-qPCR
Os02g0589400-R	ACATATAATGGCAGTCGCCGTA	RT-qPCR
Os02g0214300-F	GCCAGAGTATCTTGGCCTCC	RT-qPCR
Os02g0214300-R	AAAGCAATCTCAGGCACCGT	RT-qPCR
actin-F	GAGTATGATGAGTCGGGTCCAG	RT-qPCR
actin-R	ACACCAACAATCCCAAACAGAG	RT-qPCR
